# Atheroma-Relevant 7-Oxysterols Differentially Upregulate Cd14 Expression

**DOI:** 10.3390/ijms241310542

**Published:** 2023-06-23

**Authors:** Bo-Young Kim, Yonghae Son, Byoung Joon Kim, Sung Woon Chung, Dongjun Lee, Seong-Kug Eo, Koanhoi Kim

**Affiliations:** 1 Department of Pharmacology, School of Medicine, Pusan National University, Yangsan 50612, Republic of Korea; kimboyoung@pusan.ac.kr (B.-Y.K.); squall0211@hanmail.net (Y.S.); 2 Kim Byoung Joon Ledas Varicose Vein Clinic, Busanjin-gu, Busan 47256, Republic of Korea; 3 Department of Thoracic and Cardiovascular Surgery, School of Medicine, Pusan National University, Busan 49241, Republic of Korea; sungwoon@pusan.ac.kr; 4 Department of Convergence Medicine, School of Medicine, Pusan National University, Yangsan 50612, Republic of Korea; lee.dongjun@pusan.ac.kr; 5 College of Veterinary Medicine and Bio-Safety Research Institute, Jeonbuk National University, Iksan 54596, Republic of Korea; vetvirus@chonbuk.ac.kr

**Keywords:** CD14, inflammation, THP-1 cells, MMP-9, 7-oxysterols

## Abstract

The expression of CD14 in monocytic cells is elevated in atherosclerotic lesions where 7-oxyterols are abundant. However, it remains unknown whether atheroma-relevant 7-oxysterols are involved in receptor expression. Therefore, we investigated the effects of 7α-hydroxycholesterol (7αOHChol), 7β-hydroxycholesterol (7βOHChol), and 7-ketocholesterol (7K) on CD14 levels in THP-1 cells. The three 7-oxysterols increased CD14 transcript levels at a distinct time point, elevated cellular CD14 protein levels, and promoted the release of soluble CD (sCD14) from THP-1 cells. Our data revealed that CD14 expression was most strongly induced after treatment with 7αOHChol. Moreover, 7αOHChol alone upregulated membrane-bound CD14 levels and enhanced responses to lipopolysaccharides, as determined by CCL2 production and monocytic cell migration. The 7-oxysterols also increased the gelatinolytic activity of MMP-9, and a cell-permeable, reversible MMP-9 inhibitor, MMP-9 inhibitor I, significantly impaired sCD14 release. These results indicate that 7-oxysterols differentially induce CD14 expression in vascular cells and contribute to the monocytic cell expression of CD14 via overlapping, but distinct, mechanisms.

## 1. Introduction

The 7-carbon atom of cholesterol is easily oxygenated under oxidative stress by elevated intracellular levels of reactive oxygen species (ROS) and 7-oxygenated cholesterol molecules (7-oxysterols). For example, 7α-hydroxycholesterol (7αOHChol), 7β-hydroxycholesterol (7βOHChol), and 7-ketocholesterol (7K) are generated by the non-enzymatic oxidation of cholesterol [1,2]. Moreover, 7αOHChol is also formed by cholesterol 7α-hydroxylase (CYP7A1), an oxidoreductase located in the endoplasmic reticulum [2]. These molecules are involved in cell death, lipid metabolism, cell differentiation, and inflammation [3]; they downregulate cholesterol production by inhibiting 3-hydroxy-3-methylglutaryl-CoA reductase [4]. However, they have differential effects on immune cells; 7αOHChol induces monocytic cell differentiation into dendritic cells [5], whereas 7K stimulates macrophage differentiation [6]. 7αOHChol increases the production of chemokines, thereby enhancing the migration of immune cells such as monocytic cells and T cells that express CCR5 [7,8]. It also upregulates the expression of toll-like receptor (TLR)-6 and promotes the response to FSL-1, which activates the TLR2/TLR6 heterodimer [9]. These findings suggest distinctly overlapping biological effects of the 7-oxysterols.

Myelomonocytic cell lineages, such as monocytes and macrophages, express CD14 [10]. CD14 exists as a membrane-bound (mCD14) and soluble (sCD14) protein. The membrane-bound form is immobilized on the cell surface by a glycosylphosphatidylinositol tail, and sCD14 is either secreted from intracellular vesicles or released upon the enzymatic cleavage of mCD14 [11]. The receptor binds to and presents lipopolysaccharides (LPS), the major component of the outer membrane of gram-negative bacteria, and TLR-4, culminating in the innate immune response to bacterial LPS [12,13]. In addition, it recognizes lipoteichoic acid, a major component of the cell walls of gram-positive bacteria, and binds to minimally modified low-density lipoprotein [14,15]. The monocytes/macrophages play a central role in atherosclerosis, an inflammatory disease [16]. Notably, CD14 is upregulated in thrombotic carotid lesions, and macrophage expression of CD14 is associated with complicated lesions [17]. Since CD14 is an activator of the innate immune system, its expression is likely to correlate with inflammatory responses in atherosclerosis. However, the molecules that upregulate CD14 expression in the vasculature are poorly defined.

The second-most abundant oxysterol species in atherosclerotic lesions, 7-oxysterols, is believed to play an active role in plaque development [1]. Although it is well documented that atheroma-relevant 7-oxysterols cause cell death and control cholesterol homeostasis [3,18], whether they are involved in immune responses remains to be clarified. We assumed that endogenous molecules in atherosclerotic lesions could influence CD14 expression in monocytes/macrophages and activate the inflammatory immune response to bacterial components. Therefore, we investigated the effects of 7-oxysterols on CD14 expression in conjunction with matrix metalloproteinase (MMP)-9 and LPS responses at a concentration that did not change the viability of the THP-1 cells.

## 2. Results

### 2.1. Differential Upregulation of CD14 in the Presence of 7-Oxysterols

**Figure 1:**
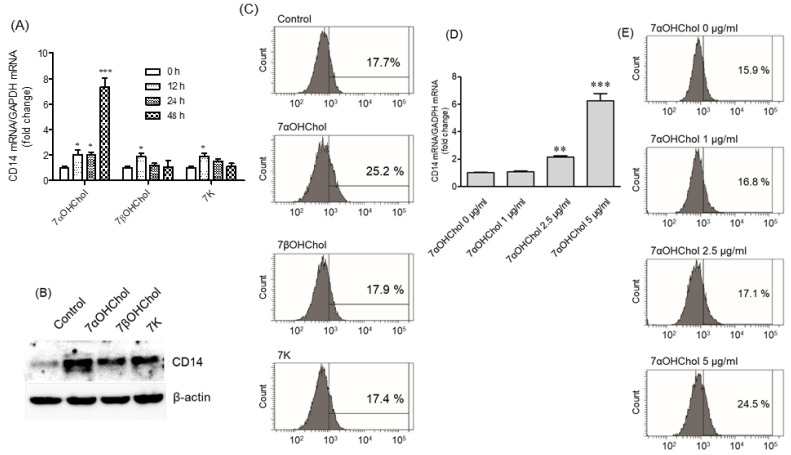
The effects of 7-oxysterols on CD14 expression at the mRNA and protein levels. THP-1 cells were serum-starved for 24 h in RPMI 1640 containing 0.1% endotoxin-free BSA, and then incubated with or without 7αOHChol, 7βOHChol, or 7K (5 μg/mL each) for the indicated periods. (**A**) CD14 transcript levels were assessed by qPCR. *Y*-axis values represent fold increases in CD14 mRNA levels normalized to GAPDH levels compared with those of control cells incubated without oxygenated cholesterol derivatives. Data are expressed as mean ± SD (n = 3 replicates/group). * *p* < 0.05 vs. control; *** *p* < 0.001 vs. control. (**B**) CD14 protein was detected by Western blot analysis, and (**C**) levels of mCD14 were measured by flow cytometry. The results are representative of three independent experiments. THP-1 cells were incubated with the indicated concentrations of 7αOHChol for 48 h after serum starvation. (**D**) The levels of CD14 transcripts were assessed by qPCR. Data are expressed as the mean ± SD (n = 3 replicates/group). ** *p* < 0.01 vs. control; *** *p* < 0.001 vs. control. (**E**) Levels of mCD14 were determined by flow cytometry. BSA: bovine serum albumin; 7αOHChol: 7α-hydroxycholesterol; 7βOHChol: 7β-hydroxycholesterol; 7K: 7-ketocholesterol; SD: standard deviation.

We determined whether the 7-oxysterols detected in atherosclerotic lesions affected CD14 expression in THP-1 cells by investigating the time-course effects of 7αOHChol, 7βOHChol, and 7K via qPCR and RT-PCT (Figure 1A, Supplementary Materials Figure S1). The mRNA levels of CD14 increased 12 h post-treatment with 7αOHChol, and further increased at 48 h post-treatment. Treatment with 7βOHChol and 7K also increased the levels of the CD14 transcripts 12 h post-treatment, which was reduced to the basal level at 48 h post-treatment. Of the three oxysterols, 7αOHChol most significantly increased the CD14 transcript levels. Next, we investigated the effects of oxysterols on CD14 protein. Treatment with 7αOHChol, 7βOHChol, and 7K increased the cellular CD14 protein levels (Figure 1B). However, flow cytometry findings revealed that the levels of mCD14 were upregulated on the surfaces of THP-1 cells incubated with 7αOHChol, but not with 7βOHChol or 7K (Figure 1C). Collectively, these results indicate that 7-oxysterols differentially enhance CD14 expression in THP-1 cells, and 7αOHChol was the strongest inducer of CD14 expression among the 7-oxysterols.

We next determined the effects of 7αOHChol at different concentrations. While 7αOHChol slightly affected the expression level of CD14 at 1 µg/mL, the levels of CD14 transcripts increased at 2.5 µg/mL 7αOHChol. This was further enhanced at 5 µg/mL 7αOHChol, as determined by quantitative PCR (Figure 1D). The level of mCD14 was also upregulated by 5 µg/mL 7αOHChol (Figure 1E). These results indicate that the levels of CD14 increase with increases in the concentration of 7αOHChol.

### 2.2. Involvement of MMP-9 in sCD14 Release following Incubation with 7-Oxysterols

**Figure 2:**
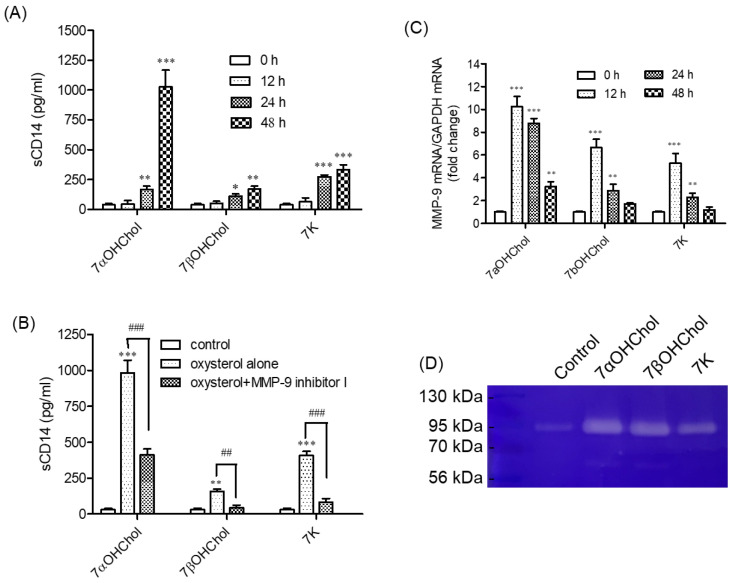
The effects of 7-oxysterols on sCD14 formation and MMP-9 activation. (**A**) Serum-starved THP-1 cells were incubated with or without 7αOHChol, 7βOHChol, or 7K (5 μg/mL each) for the indicated time periods. The levels of sCD14 in the culture media were determined by ELISA. Data are expressed as mean ± SD (n = 3 replicates/group). * *p* < 0.05 vs. 0 h; ** *p* < 0.01 vs. 0 h; *** *p* < 0.001 vs. 0 h. (**B**) Serum-starved THP-1 cells were incubated with the indicated 7-oxysterols with or without MMP-9 inhibitor I for 48 h, after which sCD14 levels in the culture media were measured by ELISA. Data are expressed as mean ± SD (n = 3 replicates/group). ** *p* < 0.01 vs. control; ***p < 0.001 vs. control; ^##^ *p* < 0.01 vs. oxysterol+MMP-9 inhibitor I; ^###^ *p* < 0.001 vs. oxysterol+MMP-9 inhibitor I. After serum starvation, THP-1 cells were incubated with or without 7αOHChol, 7βOHChol, or 7K (5 μg/mL each) for the indicated time periods. (**C**) MMP-9 transcript levels were assessed using qPCR. Data are expressed as mean ± SD (n = 3 replicates/group). ** *p* < 0.01 vs. 0 h; *** *p* < 0.001 vs. 0 h. (**D**) The activity of MMP-9 secreted by the cells was assessed by gelatin zymography. Control cells were cultured for 48 h in a medium without additives. 7αOHChol: 7α-hydroxycholesterol; 7βOHChol: 7β-hydroxycholesterol; 7K: 7-ketocholesterol; sCD14: soluble CD14; ELISA: enzyme-linked immunosorbent assay; SD: standard deviation.

We investigated whether 7-oxysterols affect sCD14 formation (Figure 2A). THP-1 cells constitutively released basal levels of sCD14, which increased in proportion along with the duration of incubation with 7-oxysterols. The levels of sCD14 in the media increased after 24 h of treatment with 7αOHChol, 7βOHChol, or 7K, and this increase was enhanced up to 48 h post-treatment. 7αOHChol most significantly promoted the formation of sCD14. We investigated whether MMP-9 participated in 7-oxysterol-induced secretion of sCD14, because MMP-9 cleaves mCD14 to release sCD14. We used a cell-permeable, selective, and reversible inhibitor of MMP-9 (MMP-9 inhibitor I; Figure 2B). The inhibitor significantly attenuated the release of sCD14 induced by 7αOHChol, 7βOHChol, and 7K. These results suggest that MMP-9 participates in sCD14 formation by 7-oxysterols.

### 2.3. Activation of MMP-9 Proteolytic Activity following Treatment with 7-Oxysterols

As the inhibition of MMP-9 resulted in an impaired release of sCD14, we investigated whether 7-oxysterols upregulated MMP-9 activity. MMP-9 transcript levels increased at various time points after incubating the cells with 7αOHChol, 7βOHChol, and 7K (Figure 2C). MMP-9 transcript levels increased 12 h after treatment with 7αOHChol, and this increase was sustained for up to 48 h after treatment. Both 7βOHChol and 7K also increased MMP-9 transcripts at 12 h post-treatment, which was sustained for up to 24 h and then decreased to basal levels at 48 h post-treatment. We also investigated the activity of the secreted MMP-9 (Figure 2D). Gelatin zymography revealed that incubation with 7αOHChol, 7βOHChol, and 7K increased MMP-9 activity. These results indicate that 7-oxysterols induce the expression of MMP-9 and increase the secretion of its active form from THP-1 cells.

### 2.4. Enhanced Responses to LPS in the Presence of 7αOHChol

**Figure 3:**
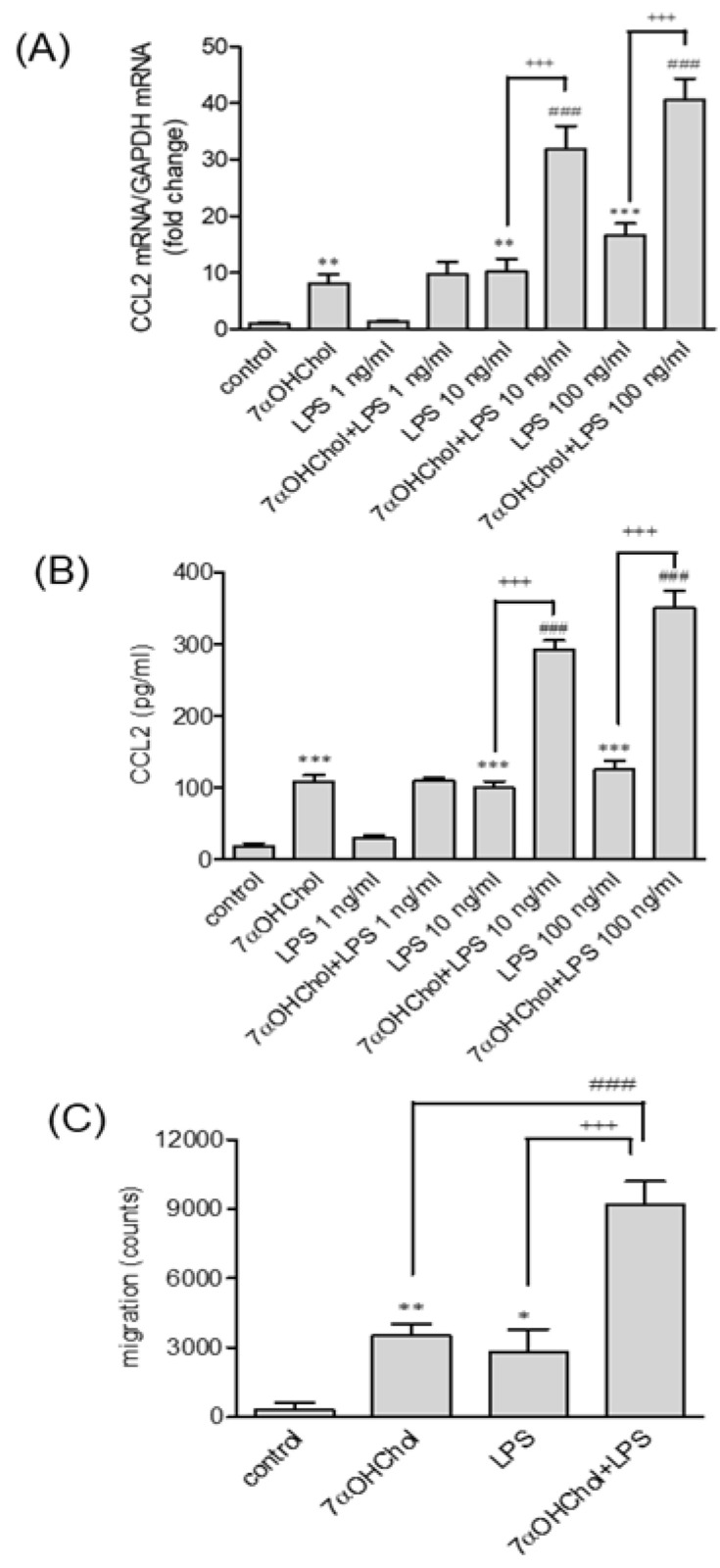
The dose effects of LPS in the presence of 7αOHChol. Serum-starved THP-1 cells were incubated for 24 h with or without 7αOHChol, and then stimulated for 9 h with the indicated concentrations of LPS from *Escherichia coli* K12. (**A**) CD14 transcript levels were assessed by qPCR. The expression levels were normalized to GAPDH levels, compared with those of control cells. Data are expressed as mean ± SD (n = 3 replicates/group). (**B**) The amount of CCL2 in the culture media was determined by ELISA (**B**). Data are expressed as mean ± SD (n = 3 replicates/group). ** *p* < 0.01 vs. control; *** *p* < 0.001 vs. control; ^###^ *p* < 0.001 vs. 7αOHChol; ^+++^ *p* < 0.001 vs. LPS. (**C**) THP-1 cells were incubated with the supernatants prepared from THP-1 cells stimulated with 7αOHChol, LPS, or 7αOHChol plus LPS. Cell migration was measured using chemotaxis assays. Data are expressed as mean ± SD (n = 3 replicates/group). * *p* < 0.05 vs. control; ** *p* < 0.01 vs. control; ^###^ *p* < 0.001 vs. 7αOHChol; ^+++^ *p* < 0.001 vs. LPS. 7αOHChol: 7α-hydroxycholesterol; LPS: lipopolysaccharide; SD: standard deviation; ELISA: enzyme-linked immunosorbent assay.

Since we found that 7αOHChol significantly upregulated both mCD14 and sCD14 levels, we investigated whether it influenced the responses to LPS. We determined the dose-dependent effects of LPS combined with 7αOHChol, and found that the CCL2 transcription-induced LPS, or 7αOHChol alone, was further induced in the presence of both LPS and 7αOHChol. Final concentrations of 10 and 100 ng/mL LPS added to cells incubated with 7αOHChol led to enhanced CCL2 transcription compared with either of them alone (Figure 3A). In contrast, adding LPS to 7βOHChol or 7K did not enhance CCL2 transcript levels compared to LPS (Supplementary Materials Figure S2). We also found that CCL2 was secreted following a pattern similar to that of gene transcription (Figure 3B). Both 7αOHChol and LPS induced CCL2 secretion. The addition of LPS at final concentrations of 10 and 100 ng/mL resulted in enhanced CCL2 secretion from cells incubated with 7αOHChol. We determined the functionality of the secreted CCL2 using chemotaxis assays (Figure 3C). Monocytic cell migration increased in response to culture supernatants isolated after stimulation with either 7αOHChol or LPS, and the migration was further increased in response to the supernatants collected from cells stimulated with 7αOHChol plus LPS. We also investigated whether the oxysterol affected TLR4 expression. The TLR4 transcript levels were not changed by the 7αOHChol treatments, as assessed by qPCR (Supplementary Materials Figure S3). These results suggest that 7αOHChol intensifies the inflammatory response to LPS.

## 3. Discussion

The CD14 pattern recognition receptor is implicated in multiple biological functions, such as monocyte activation, leukocyte–endothelial cell interactions, and apoptosis [19,20,21]. As receptor expression is a predictor of inflammation and atherosclerosis, determining the cholesterol derivatives responsible for its expression is important in vascular diseases. To investigate the effects of 7-oxysterols on the vascular cell expression of CD14, we analyzed its expression in human aortic smooth muscle cells, THP-1 monocyte/macrophage cells, and Jurkat T cells after treatment with 7αOHChol, 7βOHChol or 7K, and identified the cell types that responded to the treatments (Supplementary Materials Figure S4). Low levels of CD14 transcripts were detected by RT-PCR in the three cell types, and CD14 expression was not increased in human aortic smooth muscle cells or Jurkat cells by the 7-oxysterols tested herein. However, the data revealed significantly elevated CD14 transcript levels in THP-1 cells incubated with 7αOHChol (Supplementary Materials Figure S4A,B). These results suggest that monocytes/macrophages are the major cell types whose CD14 levels are altered in response to oxysterols; this is consistent with previous observations of CD14 upregulation in CD68-positive macrophages in atherosclerotic plaques and monocytic cells, but not in vascular smooth muscle cells or T cells, in response to 27-hydroxycholesterol [17,22].

We compared CD14 expression profiles at the mRNA and protein levels after incubating THP-1 cells with 7-oxysterols, and found that all of them showed elevated CD14 transcript levels within 12 h post-treatment. While the elevation in expression induced by 7βOHChol or 7K decreased thereafter, CD14 gene transcription induced by 7αOHChol was further enhanced for up to 48 h post-treatment. The 7-oxysterols also upregulated the total amount of CD14 protein and increased sCD14 release via MMP-9 for up to 48 h, but 7αOHChol alone induced the upregulation of mCD14 in THP-1 cells. Collectively, these results suggest that 7-oxysterols differentially upregulate the expression of CD14 transcripts and mCD14 in THP-1 cells with distinct time courses.

The multi-ligand pattern recognition receptor CD14 recognizes and binds to different pathogen- and danger-associated molecular patterns (PAMPs and DAMPs) [23]. The innate immune system detects LPS, a bacterial PAMP, and triggers pro-inflammatory pathways via TLR signaling complexes [24]. As CD14 interacts with the LPS-binding protein (LBP) and acts as a co-receptor with TLR-4 and MD2 for LPS detection [13], its expression is crucial for the innate immune response to LPS. The results of increased CCL2 production and monocytic cell migration indicated that 7αOHChol enhanced the inflammatory response to LPS by elevating mCD14 expression on THP-1 cells; 7αOHChol is abundant in atherosclerotic plaques [1]. These results, together with previous findings, suggest that 7αOHChol contributes to the development of atherosclerotic plaques by inducing inflammation and complicating the immune response upon infection with gram-negative bacteria.

sCD14 is directly secreted from intracellular vesicles or is formed by the cleavage of mCD14 by enzymes such as MMP-9 [11,25]. We showed that 7-oxysterols increased the proteolytic activity of MMP-9, and its pharmacological inhibition resulted in significant suppression of sCD14 formation, suggesting that 7-oxysterol enhances the cleavage of mCD14 on THP-1 cells via MMP-9. However, the inhibition did not completely suppress sCD14 formation. Therefore, 7-oxysterols may activate other mechanisms of CD14 formation, such as cleavage by other proteases, including MMP-12, or secretion from vesicles [26]. We believe that the secreted sCD14 is likely to influence inflammation. sCD14 enables endothelial cells to respond to LPS [27], which triggers apoptosis in endothelial cells through an sCD14-dependent mechanism [20]. Moreover, sCD14 binds membrane proteins or phospholipids that may be responsible for the production of inflammatory cytokines [28]; induces pro-inflammatory cytokine production via TLR-4 in the immune cells of autoimmune diseases [29]; and acts as a DAMP to activate macrophages in infectious inflammatory diseases [24]. Therefore, we suggest that 7-oxysterols may predispose atherosclerotic lesions to innate immune responses against pathogens that contain receptor ligands.

Compared to normal arteries in which pro-MMP-2 and tissue inhibitors of metalloproteinases are expressed without detectable MMP activity, the levels of multiple proteases, including MMP-9, are elevated in atheromatous plaques [30,31]. Matrix metallopeptidases directly degrade extracellular matrix proteins and promote rupture by weakening plaque caps; however, MMP-9 overexpression is insufficient to degrade the matrix component elastin because macrophages predominantly secrete inactive pro-MMP-9. The expression of activated MMP-9 induces acute plaque disruption in apoE-deficient mice [32]. We observed the release of active MMP-9 from THP-1 cells in an environment rich in 7-oxysterols, which agreed with the fact that macrophages are the primary cell source for MMP-9 activity in atherosclerotic plaques [33]. Taken together, the present findings suggest that 7-oxysterols contribute to plaque rupture by increasing the proteolytic activity of MMP-9. MMP-9 is converted to its active form following enzymatic proteolysis of its pro-domain [33,34]. However, this study did not determine post-activation processing, and it is unknown how 7-oxysterols affect the enzymatic cleavage of pro-MMP-9. The mechanisms underlying MMP-9 activation in the presence of 7-oxysterols are currently under investigation.

**Figure 4:**
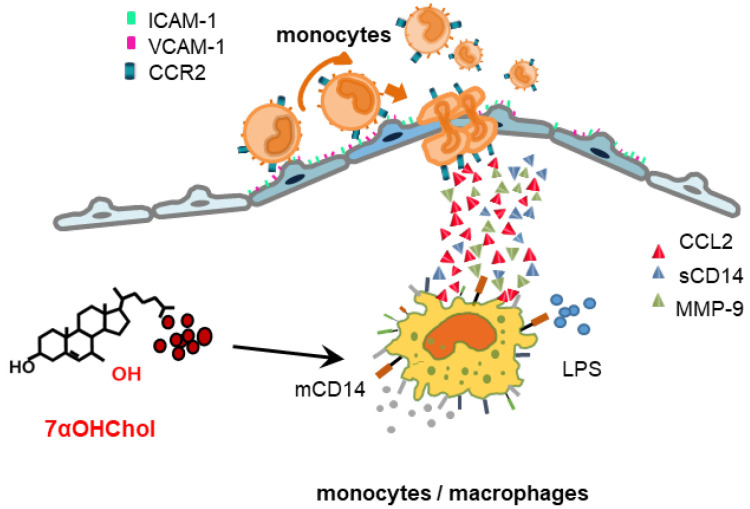
Pro-inflammatory activity of 7αOHChol in atherosclerotic lesions. 7αOHChol activates monocytic cells and leads to the upregulation of mCD14 and the secretion of active MMP-9 in the lesions. Active enzymes promote sCD14 formation. The increase in CD14 expression enhances responses to LPS and causes the further secretion of CCL2 chemokines from monocytic cells. The secreted chemokines stimulate the migration of monocytic cells attached to the activated endothelium. 7αOHChol: 7α-hydroxycholesterol; mCD14: membrane-bound CD14; sCD14: soluble CD14; LPS: lipopolysaccharide.

The results of this study, along with those of previous studies, suggest a model of inflammation via which 7αOHChol contributes to plaque development in atherosclerosis (Figure 4). It activates monocytic cells to secrete CCL2 and MMP-9 and to increase the levels of mCD14. CCL2 enhances the migration of CCR2-positive immune cells attached to the endothelium, which expresses cell adhesion molecules, and MMP-9 is activated to cleave mCD14 to sCD14. Increased mCD14 and sCD14 levels result in the delivery of LPS to TLR4 after infection with gram-negative bacteria, which further affects the secretion of CCL2 and enhances the migration of immune cells. Active MMP-9 also degrades the extracellular matrix component and promotes plaque instability.

In addition to the induction and activation of MMP-9 and its involvement in sCD14 formation in a milieu rich in 7-oxysterols, we demonstrated that 7-oxysterols differentially upregulate mCD14 levels. These new findings raise questions regarding the molecular mechanisms underlying the differential upregulation of mCD14 among the 7-oxysterols, as well as the biological functions of sCD14 formed by 7-oxysterols and MMP-9 activation. These questions should be addressed in future studies in order to better understand the pathophysiological roles of 7-oxysterols in inflammation associated with atherosclerosis by using human primary cells and in vivo models.

## 4. Materials and Methods

### 4.1. Cells and Reagents

The human THP-1 monocytic cell line was purchased from the American Type Culture Collection (ATCC) (Manassas, VA, USA). The cells were cultured and maintained at a cell density between approximately 2.0 × 10^5^ and 8.0 × 10^5^ viable cells/mL, as recommended by ATCC; 7αOHChol, 7βOHChol, and 7K were purchased from Research Plus, Inc. (Barnegat, NJ, USA). MMP-9 inhibitor I and LPS prepared from *Escherichia coli* K12 were purchased from Millipore Sigma (Burlington, MA, USA) and InvivoGen (San Diego, CA, USA), respectively. Antibodies against CD14 and β-actin were purchased from Santa Cruz Biotechnology, Inc. (Dallas, TX, USA).

### 4.2. Real-Time Polymerase Chain Reaction (qPCR)

Total RNA was reverse-transcribed for 1 h at 42 °C using Moloney murine leukemia virus reverse transcriptase (Promega, Madison, WI, USA), and qPCR was performed in triplicate using a LightCycler^®^ 96 Real-Time PCR System (Roche Holdings AG, Basel, Switzerland). The reactions (20 μL) contained 4 μL of cDNA template, 10 μL of SYBR Green Master Mix, and 2 μL of 10 pM forward and reverse primers of the target gene, which was amplified under the following cycling conditions: 95 °C for 10 min and 45 cycles at 95 °C for 10 s, 50 °C for 10 s, and 72 °C for 10 s. Gene expression was calculated relative to that of the housekeeping gene, glyceraldehyde-3-phosphate dehydrogenase (GAPDH), using LightCycler^®^ 96 software (Version 1.1.0.1320, Roche), and the mRNA levels of the target genes were normalized to those of glyceraldehyde-3-phosphate dehydrogenase (GAPDH) using the 2^−ΔΔCt^ method [35]. The forward and reverse (5′→3′) primers were

CD14, F: ACGCCAGAACCTTGTGAGC, and R: GCATGGATCTCCACCTCTACTG:

CCL2, F: CAGCCAGATGCAATCAATGCC, and R: TGGAATCCTGAACCCACTTCT;

MMP-9, F: GCACGACGTCTTCCAGTACC, and R: CAGGATGTCATAGGTCACGTAGC;

GAPDH, F: GAAGGTGAAGGTCGGAGT, and R: GAAGATGGTGATGGGATTTC.

### 4.3. Flow Cytometry

Following treatment with 7αOHChol, 7βOHChol, and 7K for 48 h, THP-1 cells were washed with phosphate-buffered saline (PBS) containing 0.5% bovine serum albumin (BSA) and 2 mM ethtlene diamine tetra acetic acid (EDTA) (washing buffer), then incubated with 3% BSA in phosphate-buffered saline (PBS). The cells were exposed to anti-CD14 antibody diluted 1:100 in washing buffer at 4 °C overnight. The cells were washed three times and incubated with anti-mouse IgG-Alexa Fluor 488 diluted 1:200 in washing buffer for 40 min at room temperature in the dark. Fluorescence emission by the stained cells was analyzed using a FACSCanto^TM^ II flow cytometer (BD Biosciences, Franklin Lakes, NJ, USA).

### 4.4. Enzyme-Linked Immunosorbent Assay

The levels of sCD14 and CCL2 secreted from the cells into the culture media were measured using ELISA kits, according to the manufacturer’s instructions (BD Bioscience, Franklin Lakes, NJ, USA). After serum starvation with 0.1% BSA in RPMI 1640 medium overnight, THP-1 cells were exposed to oxysterols. The cells were centrifuged (200× *g*; 5 min), and the supernatants were collected and frozen at −20 °C. The supernatants, which were thawed prior to use, and standards for CCL2 or sCD14 were added to a microtiter plate pre-coated with a monoclonal antibody against CCL2 or sCD14, followed by incubation for 1 h. Then, the wells were washed and incubated with an enzyme-conjugated antibody for each molecule. The substrate provided in the kit was added after three washes, and the color intensity was measured using a Sunrise microplate reader (Tecan Austria GMBH, Grödig, Austria). The amounts of CCL2 and sCD14 were determined using a standard curve.

### 4.5. Western Blot Analysis

Cell lysates were separated using 10% sodium dodecyl sulfate-polyacrylamide gel electrophoresis and transferred to nitrocellulose membranes. Non-specific protein binding was blocked for 1 h in Tris-buffered saline (TBS) containing 0.05% Tween-20 (TBS-T) and 1% skimmed milk, and the membranes were incubated with primary antibodies at 4 °C overnight. After three washes with TBS-T, the membranes were incubated for 1 h with horseradish peroxidase (HRP)-conjugated secondary antibodies at room temperature. Bands were detected using chemiluminescent reagents. Chemiluminescent images were captured using an Amersham Imager 600 (GE Healthcare Life Sciences, Pittsburgh, PA, USA).

### 4.6. Migration Assay

Cell migration was investigated using Transwell^TM^ Permeable Supports (Costar, Cambridge, MA, USA) as previously described [8]. The top wells of 5-micrometer-pore polycarbonate Transwell^TM^ inserts loaded with monocytic cells were inserted into reservoirs containing supernatants with chemoattractants. After 3 h in a CO_2_ incubator, the number of cells that migrated into the reservoir was counted using a Vi-Cell XR cell counter (Beckman Coulter, Life Sciences Division, Indianapolis, IN, USA).

### 4.7. MMP-9 Gelatinolytic Activity in Cell Supernatants

MMP-9 activity was assessed using gelatin zymography as previously described [36]. After serum-free incubation of THP-1 cells with or without the indicated 7-oxysterols, the supernatants were collected after centrifugation (200× *g*; 5 min) and concentrated 30-fold using a Vivaspin^®^ 2 concentrator (Sartorius Lab Instruments AG., Göttingen, Germany). The concentrated supernatants were electrophoretically separated on 8% polyacrylamide gels containing 0.15% gelatin. The gels were washed, activated for 18 h at 37 °C, stained with 0.2% Coomassie Brilliant Blue R-250, and destained with 20% methanol/10% acetic acid. The bands in the gels were visualized using ZoomBrowser EX 5.0 (Canon, Tokyo, Japan).

### 4.8. Statistical Analysis

The data were statistically analyzed by one-way analysis of variance (ANOVA), followed by Dunnett’s multiple comparison tests, using PRISM (version 5.0; GraphPad Software Inc., San Diego, CA, USA). A *p*-value less than 0.05 was considered statistically significant.

## Data Availability

All data generated or analyzed during this study are included in this published article and its supplementary information files.

## Supplementary Materials

The following supporting information can be downloaded at: https://www.mdpi.com/article/10.3390/ijms241310542/s1.
