# Supplementary material for: Atheroma-Relevant 7-Oxysterols Differentially Upregulate Cd14 Expression
Source: Int J Mol Sci. 2023 Jun 23;24(13):10542. doi: 10.3390/ijms241310542 (PMC10341412; doi:10.3390/ijms241310542)
Supplement: Supplementary file 1 [file ijms-24-10542-s001.zip › ijms-2430655-supplementary.pdf]

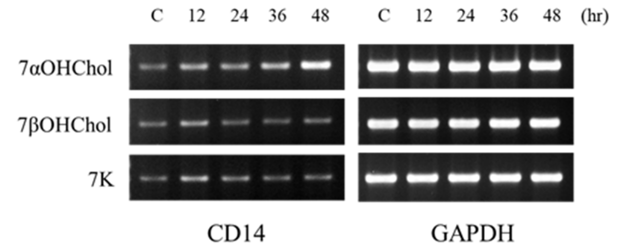

Figure S1. Time course expression of CD14 mRNA in THP-1 cells after treatment with the indicated 7-oxysterols. THP-1 cells were serum-starved for 24 h in RPMI 1640 containing 0.1% endotoxin-free BSA, and then incubated with or without 7αOHChol, 7βOHChol or 7K (5 μg/mL each) for the indicated time periods. CD14 transcripts were amplified by RT-PCR. PCR products were separated on 2% agarose gels and stained with ethidium bromide. BSA: bovine serum albumin; 7αOHChol: 7α-hydroxycholesterol; 7βOHChol: 7β-hydroxycholesterol; 7K: 7-ketocholesterol; GAPDH: glyceraldehyde-3-phosphate dehydrogenase.

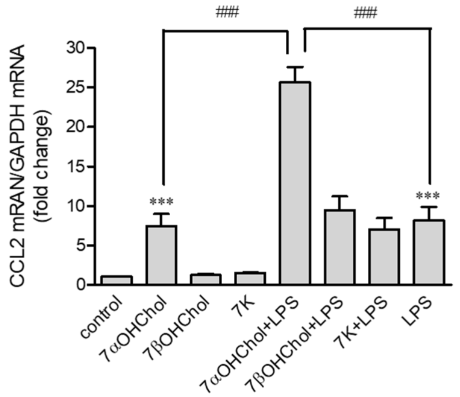

Figure S2. CCL2 expression in the THP-1 cells stimulated with LPS in presence of 7-oxysterols. Serum-starved THP-1 cells were incubated for 24 h with the indicated 7-oxysterols (5 μg/mL each), and then stimulated for 9 h with or without LPS (100 ng/mL) from *Escherichia coli* K12. CCL2 transcript levels were assessed by real-time PCR. Data are expressed as mean ± SD (n = 3 replicates/group). $^{***}P < 0.001$ vs. control; $^{\#\#\#}P < 0.001$ vs. 7αOHChol or LPS. 7αOHChol: 7α-hydroxycholesterol; 7βOHChol: 7β-hydroxycholesterol; 7K: 7-ketocholesterol; LPS: lipopolysaccharide; SD: standard deviation.

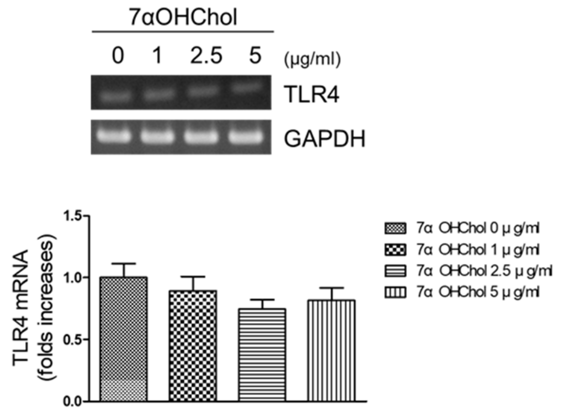

Figure S3. Effects of 7αOHChol on expression of TLR4 in THP-1 cells. THP-1 cells were serum-starved for 24 h in RPMI 1640 containing 0.1% endotoxin-free BSA, and then incubated with the indicated concentration of 7αOHChol for 48 h. TLR4 transcripts were analyzed by RT-PCR and assessed by quantitative real-time PCR. These results were derived from three independent experiments. 7αOHChol: 7α-hydroxycholesterol; TLR4: Toll-like receptor 4.

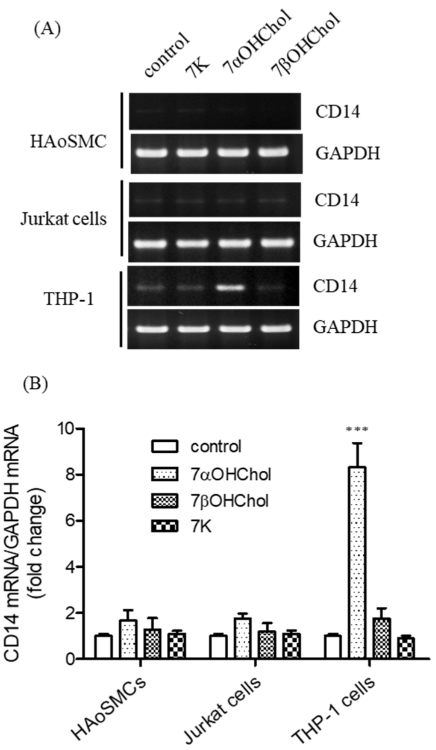

Figure S4. The effects of 7-oxysterols on the CD14 expression in vascular cells in the atherosclerotic lesions. human aortic smooth muscle cells (HAoSMCs), Jurkat T cells, and THP-1 monocytes/macrophages were serum-starved for 24 h in RPMI 1640 containing 0.1% endotoxin-free BSA, and then incubated for 48 h with or without 7αOHChol, 7βOHChol or 7K (5 μg/mL each). (A) CD14 mRNA was amplified by RT-PCR. PCR products were separated on 2% agarose gels and stained with ethidium bromide. (B) CD14 transcript levels were assessed by RT-PCR. Y-axis values represent fold increases in CD14 mRNA levels normalized to those of control cell types incubated without an oxygenate cholesterol molecule. Data are expressed as mean ± SD (n = 3 replicates/group). ***$P < 0.001$ vs. control. BSA: bovine serum albumin; 7αOHChol: 7α-hydroxycholesterol; 7βOHChol: 7β-hydroxycholesterol; 7K: 7-ketocholesterol; GAPDH: glyceraldehyde-3-phosphate dehydrogenase.
